# Comparison of DNA Extraction Methods for Microbial Community Profiling with an Application to Pediatric Bronchoalveolar Lavage Samples

**DOI:** 10.1371/journal.pone.0034605

**Published:** 2012-04-13

**Authors:** Dana Willner, Joshua Daly, David Whiley, Keith Grimwood, Claire E. Wainwright, Philip Hugenholtz

**Affiliations:** 1 Australian Centre for Ecogenomics, School of Chemistry and Molecular Biosciences and Institute of Molecular Bioscience, The University of Queensland, St. Lucia, Queensland, Australia; 2 Diamatina Institute, The University of Queensland, St. Lucia, Queensland, Australia; 3 Queensland Paediatric Infectious Diseases Laboratory, Infection Management and Prevention Service, Royal Children's Hospital, Brisbane, Queensland, Australia; 4 Queensland Children's Medical Research Institute, Royal Children's Hospital, The University of Queensland, St. Lucia, Queensland, Australia; 5 Queensland Children's Respiratory Centre, Royal Children's Hospital, Herston, Queensland, Australia; Cairo University, Egypt

## Abstract

Barcoded amplicon sequencing is rapidly becoming a standard method for profiling microbial communities, including the human respiratory microbiome. While this approach has less bias than standard cultivation, several steps can introduce variation including the type of DNA extraction method used. Here we assessed five different extraction methods on pediatric bronchoalveolar lavage (BAL) samples and a mock community comprised of nine bacterial genera to determine method reproducibility and detection limits for these typically low complexity communities. Additionally, using the mock community, we were able to evaluate contamination and select a relative abundance cut-off threshold based on the geometric distribution that optimizes the trade off between detecting *bona fide* operational taxonomic units and filtering out spurious ones. Using this threshold, the majority of genera in the mock community were predictably detected by all extraction methods including the hard-to-lyse Gram-positive genus *Staphylococcus*. Differences between extraction methods were significantly greater than between technical replicates for both the mock community and BAL samples emphasizing the importance of using a standardized methodology for microbiome studies. However, regardless of method used, individual patients retained unique diagnostic profiles. Furthermore, despite being stored as raw frozen samples for over five years, community profiles from BAL samples were consistent with historical culturing results. The culture-independent profiling of these samples also identified a number of anaerobic genera that are gaining acceptance as being part of the respiratory microbiome. This study should help guide researchers to formulate sampling, extraction and analysis strategies for respiratory and other human microbiome samples.

## Introduction

Microbial community profiling using the 16 S rRNA gene has experienced a recent resurgence, with the advent of high-throughput amplicon sequencing facilitating large-scale culture-independent studies of environmental microbiota [1]. In particular, this method has been widely applied to human microbiomes, most notably in the human gut and skin. Recently, characterization of the human microbiome using 16 S pyrosequencing has expanded to include the respiratory tract [2]–[6]; however, the effect of DNA extraction methods on microbial community profiles has yet to be investigated. Methodological comparisons have demonstrated that DNA extraction method can be a critical parameter in studies which use amplicon pyrosequencing as well as in shotgun metagenomics [7]–[9].

Bronchoalveolar lavage samples (BAL) are considered the gold standard for sampling microbial communities in the lower respiratory tract, and have been shown to produce community profiles concordant with microbiota associated directly from lung tissue [3], [10]. BAL samples are especially useful for pediatric patients who often cannot spontaneously expectorate sputum and for whom oropharyngeal samples may not be representative of the lower airways [11], [12]. Culture-based studies have demonstrated differences in microbial communities from lavage of different lobes of the lung, while targeted molecular studies have identified differences in detection rates for specific viruses, bacteria and fungi using different DNA extraction methods and PCR assays [13]–[17]. However, methods for community profiling of pediatric BAL samples have been largely unexplored. Here, we sought to evaluate DNA extraction methods for pediatric BAL samples to determine if DNA extraction method has a significant effect on microbial community profiles. These methods were also tested on a mock community of similar complexity to model detection limits, to identify methodological contaminants, and to compare method reproducibility using a sample of known composition.

## Methods and Materials

### Ethics Statement

The BAL samples were collected as part of two different studies and approved by the Royal Children's Hospital (RCH), Brisbane Ethics Committee. Written informed consent was obtained from the children's parents for BAL sample collection, storage and subsequent testing.

### Study subjects and sample collection

Two children with cystic fibrosis (CF) who took part in the ACFBAL study and one non-CF patient recruited from the RCH provided the BAL samples used in this study. All BAL samples were collected under general anaesthesia using standard procedures [18]. A portion of the raw BAL was sent for routine microbiological culture, while the rest was frozen in aliquots at −80°C. The first CF patient was a male with two copies of the pF508del allele of the CFTR gene who was five years of age at the time of BAL collection in 2006. The second CF patient was a female who also had two copies of the pF508del allele and was four years of age at BAL collection in 2005. The non-CF patient was previously diagnosed with both tracheal dyskinesia and Down Syndrome, and was six years of age when the BAL sample used in this study was collected in 2004.

### *In vitro* mock community

A microbial community was constructed *in vitro* using twelve bacterial strains, including common microbes associated with CF and respiratory infections. Each strain was grown to a 1 McFarland standard using standard microbiological conditions and suspended in saline as follows: *Pseudomonas aeruginosa* ATCC 17503 (undiluted), *Burkholderia cepacia* ATCC 17765 (1/10 dilution), *Staphylococcus aureus* ATCC 25923 (1/10), *Haemophilus influenzae* ATCC 49247 (1/10), *Moraxella catarrhalis* ATCC 25238 (1/100), *Staphylococcus epidermidis* ATCC 14990 (1/100), *Klebsiella pneumoniae* ATCC 700603 (1/100), *Neisseria meningitidis* ATCC 13102 (1/1000), *Burkholderia multivorans* RCH clinical isolate (1/1000), *Legionella pneumophila* ATCC 33152 (1/10000), *Streptococcus pneumoniae* ATCC 49619 (1/10000), and *Neisseria gonorrhoeae* RCH clinical isolate (1/100000). Equal volumes (1.4 mL) of each suspended or re-suspended culture were added to a 50 mL tube to give a final volume of 16.8 mL. The mock community was stored at −20C prior to DNA extraction.

### DNA Extraction

DNA was extracted from 400 µL aliquots of the mock community and pediatric BAL samples using a cetyl trimethylammonium bromide (CTAB) protocol adapted from Sambrook and Russell [19], a high salt (saline) protocol adapted from Quinque et al. [20], and two commercially-available kits: the Nucleospin Tissue Kit (Macherey-Nagel, Düren, Germany) using both a pellet protocol and liquid protocol and the MoBio PowerSoil DNA Isolation Kit (MoBio Laboratories, Carlsbad, CA, US). CF BAL samples were also pre-processed with dithiothreitol (DTT) in the form of Sputasol (Oxoid, Cambridge, UK) according to the manufacturer's instructions. Aliquots of sterile water were extracted in parallel as non-template controls (NTCs) to assay for the presence of contaminants. Extracted DNA was quantified using the Qbit Fluorimeter (Invitrogen, Carlsbad, CA, US). A more detailed description of each extraction method appears below.

#### CTAB protocol

Sample aliquots were spun at 10,000×g to pellet cellular material. After removal of the supernatant, cell pellets were re-suspended in 567 µL of autoclaved and 0.2 filtered TE pH 8 and incubated for 1 hour at 37°C with 30 µL 10% sodium dodecyl sulfate (SDS) and 3 uL 20 mg/mL Proteinase K (Sigma-Aldrich, Castle Hill, NSW, Australia). Samples were then incubated for 10 minutes with 100 uL of 5 M NaCl prepared with sterile water and 80 uL of CTAB/NaCl solution (4.1 g NaCl, 10 g CTAB in 100 mL sterile water). Following incubation, extracts were purified using phenol chloroform extraction, and DNA was recovered by isopropanol precipitation. Pelleted DNA was washed twice with cold 70% ethanol, allowed to air dry, and re-suspended in 50 µL of sterile water.

#### Saline protocol

Sample aliquots were mixed with an equal volume (400 µL) of autoclaved and 0.2 µm filtered lysis buffer (50 mM Tris, pH 8.0, 50 mM EDTA, 50 mM sucrose, 100 mM NaCl, 1% SDS), 15 µL of 20 mg/mL proteinase K (Sigma-Aldrich, Castle Hill, NSW, Australia) and 75 µL of 10% SDS and incubated overnight at 56°C. Subsequently, 200 uL of 5 M NaCl was added and samples were incubated for 10 minutes on ice. Salt and cellular debris were pelleted by centrifugation at 10,000×g for 10 minutes. The supernatant was removed to a new tube and extracted DNA recovered by isopropanol precipitation. Pelleted DNA was washed twice with cold 70% ethanol, allowed to air dry, and re-suspended in 50 µL of sterile water.

#### Nucleospin Tissue Kit pellet protocol

Samples were pelleted as for the CTAB protocol above. Pellets were re-suspended in 180 µL of Buffer T1, incubated for 3 hours at 56°C with 25 µL Proteinase K in Buffer PB (20 mg/mL) and DNA extraction was carried out according to the manufacturer's protocol.

#### Nucleospin Tissue Kit liquid protocol

Samples were incubated with 25 µL of Proteinase K in Buffer PB (20 mg/mL) for 3 hours at 56°C. An equal volume (400 µL) of Buffer B3 was then added, and samples were incubated for 10 minutes at 70°C. One volume (400 µL) of 100% ethanol was added, and following vortexing, samples were loaded onto Nucleospin columns. The remainder of the extraction procedure was carried out according to the manufacturer's protocol.

#### MoBio PowerSoil Kit protocol

Sample aliquots were added directly to Powerbead tubes along with 60 µL of solution C1 and extracted according to the manufacturer's protocol.

### PCR of 16 S rRNA

The V8 and V9 regions of the 16 S rRNA gene were amplified using fusion primers containing 454 adaptor sequences ligated to the primers 1114F3-5′YAACGARCGCRACCC and 1392R-5′ACGGGCGGTGTGTRC [21]. Multiplex identifiers of 5–7 nucleotides were incorporated in the reverse primer sequence to allow for multiplexing. Duplicate 50 uL PCR reactions were prepared. Each contained 10–15 µL (mock community and water samples) or 5 uL (BAL samples) of template DNA, 5 uL of 10× buffer (Invitrogen, Carlsbad, CA, USA), 1 µL of 10 mM dNTP mix (Invitrogen, Carlsbad, CA, USA), 1.5 µL BSAI (Fermentas, CA, USA), 1.5 µL 50 mM MgCl2 (Invitrogen, Carlsbad, CA, USA), 1 µL of each 10 uM primer, and 1 unit of Taq Polymerase (Invitrogen, Carlsbad, CA, USA). Cycling conditions were one cycle of 95°C for 3 min, followed by 30 cycles of 95°C for 30 s, 55°C for 45 s and 72°C for 90 s followed by a final extension of 72°C for 10 min. Following amplification, PCR products for each sample were pooled and purified using the QIAquick PCR Purification Kit and quantitated using the Qbit Fluorimeter (Invitrogen, Carlsbad, CA, USA). Two mock community samples extracted using the PowerSoil kit, two control BAL samples, and all of the NTCs produced no detectable amplification products. The total reaction volume of four of the NTCs (CTAB, Saline, Nucleospin Pellet, and PowerSoil) was used for sequencing. Amplicons from all other samples were pooled in equal proportions. Amplicon pools were sequenced from the reverse primer using the 454 GS-FLX Titanium platform at Macrogen Inc. (Korea). 16 S sequences have been submitted to the short read archive at NCBI under BioProject ID PRJNA81021 and study ID SRA049197.1.

### Real-time PCR

Real-time PCR assays for PCR inhibition, microbial DNA and human DNA as described in [22], [23] were conducted on the control BAL samples after two samples failed to produce measurable amplification products.

### *In silico* simulation of mock BAL community

In silico libraries were created using the open-source software Grinder (http://sourceforge.net/biogrinder) to simulate 454 sequences from the *in vitro* mock community [24]. Complete chromosomal sequences of twelve microbial species were used as inputs to Grinder: *Pseudomonas aeruginosa* LESB-58 (accession number: NC_011770.1), *Burkholderia cenocepacia* HI2424 (NC_008542.1, NC_008543.1, NC_008544.1), *Staphylococcus aureus subsp. aureus* ED98 (NC_013450.1), *Haemophilus influenzae* F3047 (NC_014922.1), *Moraxella catarrhalis* RH4 (NC_014147.1), *Klebsiella pneumoniae subsp. pneumoniae* MGH 78578 (NC_009648.1), *Staphylococcus epidermidis* RP62A (NC_002976.3), *Neisseria meningitidis* Z2491 (NC_003116.1), *Burkholderia multivorans* ATCC 17616 (NC_010084.1, NC_010085.1, NC_010086.1), *Legionella pneumophila* str. Corby (NC_009494.2), *Streptococcus pneumoniae* AP200 (NC_014494.1), an*d Neisseria gonorrhoeae* FA 1090 (NC_002946.2). Relative abundances were specified to match the putative composition of the *in vitro* community as suggested by McFarland standards: approximately 75% *P. aeruginosa*, 7.5% each of *S. aureus*, *B. cenocepacia*, and *H. influenzae*, 0.75% each of *M. catarrhalis*, *K. pneumoniae*, and *S. epidermidis*, 0.075% each of *N. meningitidis* and *B. multivorans*, 0.0075% each of *L. pneumophila* and *S. pneumoniae*, and 0.00075% of *N. gonorrhoeae*. The 1114F and 1392R primer sequences were used for amplicon selection from genomic sequences with copy number bias set to true. For all libraries, reads were generated with an average length of 250 base pairs normally distributed with standard deviation of 50 base pairs. Reads were generated from the reverse primer only by specifying the unidirectional option, and five base pair multiplex identifiers were attached to the reverse primer. Homopolymer errors were introduced using the Balzer model [25], and other errors (indels and substitutions) were introduced using a linear model with frequency 0.004 at the 5′ end of reads and 0.005 at the 3′ end, according to error rates described in [26]. Three libraries containing 5000 sequences and three libraries with 100,000 sequences were generated.

### Bioinformatics

Libraries generated in silico and by 454 pyrosequencing were quality filtered and trimmed, as well as corrected for homopolymer errors using Acacia [27]. Sequences were further trimmed to a uniform length of 230 base pairs using QIIME, and any sequences less than 230 base pairs were excluded from further analysis. With the exception of the NTCs, which produced no sequences, between 400 and 8,000 sequences were obtained for each library following quality filtering (**Table S1**). Multiplexed libraries were deconvoluted and analyzed using the QIIME pipeline with taxonomy selection based on BLASTn comparison to GreenGenes (e-value<10e-5) [28], [29]. Heat maps were produced using the R package gplots [30] on data normalized to either 900 (mock community internal comparisons only) or 400 sequences (all other comparisons). Community-level analyses were performed using FastUnifrac [31]. Unifrac distances between and within DNA extraction methods and individuals were compared using the exact Mann-Whitney-U test which is appropriate for the small sample size. PERMANOVA analysis was conducted with 1000 permutations using the R package vegan [32].

The large simulated libraries were repeatedly sub-sampled using the multiple rarefaction feature in QIIME from 10 to 100 sequences at an interval of 10, from 100 to 1000 at an interval of 100, from 1000 to 10,000 at an interval of 1,000, and from 10,000 to 90,000 with an interval of 10,000 with 100 subsamples taken at each sampling point, generating a total of 300 data points [28]. Perl scripts were written to count the proportion of times each taxon was observed at each sampling level, and these proportions were used to generate an empirical cumulative distribution. The scripts can be accessed at https://sourceforge.net/projects/detthresh/. These distributions were compared to the geometric distribution which has the cumulative density function P(X≤k) = p(1−p)^k^ where k is the number of trials until the first success is observed and p is the probability of success, estimated here as the approximate operational taxonomic units (OTUs) relative abundance. Fit was assessed using bootstrapped Komologorov-Smirnov tests with 1000 repetitions to compare each empirical distribution to the corresponding cumulative geometric distribution with the taxon relative abundance used as the parameter p as implemented in the R package Matching [33].

## Results

To provide an objective comparison of DNA extraction methods, we constructed a mock microbial community comprised of taxa many of which are commonly associated with respiratory illness, and in particular, cystic fibrosis (CF) [34]. Analysis of this mock community allowed for evaluation of the technical reproducibility and efficacy of DNA extraction methods without the complicating factor of biological variation inherent in clinical samples. Extraction methods included protocols used in other studies of the human microbiome which have not previously been directly compared: a modified CTAB method [19], two variations of the Nucleospin Tissue Kit [5], the MoBio PowerSoil Kit [6], [35]–[37], and a high salt protocol [38]–[41]. A comparison of the type of extraction as well as the time and cost necessary for these methods appears in Table 1. The mock community was also simulated in silico using Grinder, a bioinformatic tool which can generate amplicon libraries with sequence lengths and error profiles characteristic of 454 pyrosequencing [24]. The five DNA extraction methods were tested on three pediatric BAL samples: two from CF patients and one from a non-CF individual with chronic respiratory disease.

**Table 1:** Comparison of DNA extraction methods.

Method	Basis	Cost per sample (AUD)	Time per sample	Advantages	Disadvantages
CTAB	Chemical/enzymatic lysis	<$5.00	∼18 hours (including overnight incubation)	Inexpensive	Many steps; overnight incubation; use of toxic chemicals such as CTAB and Phenol/Chloroform; user-made buffers may introduce contamination
Saline	Chemical/enzymatic lysis	<$2.00	∼18 hours (including overnight incubation)	Inexpensive; simple protocol with few steps and reagents	Overnight incubation; user-made buffers may introduce contamination
Nucleospin Tissue Kit	Chemical/enzymatic lysis	$5.60	3–4 hours (including 3 hour incubation)	No overnight incubation; kit method with buffers supplied; options to use pellet and liquid protocols	More expensive than non-kit methods; 1–3 hour incubation
MoBio PowerSoil Kit	Chemical/mechnical lysis	$5.50	∼1 hour	Rapid protocol; kit method with all buffers supplied; bead-beating may improve recovery for hard-to-lyse strains	More expensive than non-kit methods; difficult for large numbers of samples without special equipment (e.g. vortex adapter); multiple transfers between tubes may introduce contamination

### Detection limits of 16 S pyrosequencing in the mock community

To model the sequencing effort necessary to reliably detect microbial taxa, in silico libraries of the mock community containing 100,000 sequences each were created and repeatedly sub-sampled to construct empirical cumulative probability distributions for each taxon (Methods). The probability distributions were highly consistent with the geometric distribution, where the taxon relative abundance was used as an estimate for the parameter p (**Table S2; Figure S1A**). The number of sequences necessary to detect operational taxonomic units (OTUs) with 95% confidence was calculated as the 95^th^ percentile of the theoretical distribution (**Figure S1A&B**). According to the geometric model, more than 44,000 sequences would be necessary to detect *Streptococcus* with 95% confidence, while *Legionella* would require greater than 58,000 sequences.

**Figure 1:**
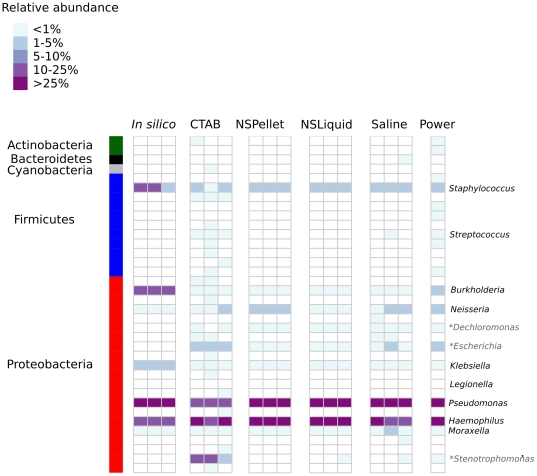
Microbial community profiles for the mock community. 16 S libraries were normalized to 900 sequences and 97% OTUs were consolidated at the genus level. The nine genera comprising the mock community are marked in black italics, while the starred genera in grey italics correspond to contaminants.

In the present study we normalized to 900 sequences per sample for which we would predict genera with estimated relative abundances less than ∼0.3% (*Legionella*, *Streptococcus* and *Neisseria*) would fall below the theoretical 95% confidence limits (**Figure S1B**). Seven of the nine component genera in the mock community were predictably detected by all extraction methods. This included the hard-to-lyse Gram-positive genus *Staphylococcus*, which was detected at the anticipated level of >1% abundance in all but one sample (CTAB replicate 2; **Figure 1**), and at nearly 10% abundance in the PowerSoil community. *Neisseria* was also observed in all sequenced samples and the in silico community, despite a predicted relative abundance less than 0.3% (**Figure 1** **; Table S2**). The model indicated that at 900 sequences the cumulative probability of detecting this genus was approximately 50%, as compared to less than 5% for *Streptococcus* and *Legionella* (**Fig. S1A**). *Legionella* was not detected in any of the pyrosequenced or in silico samples, while sequences corresponding to *Streptococcus* were present only in a small subset of pyrosequenced libraries.

### Microbial contamination in the mock community

**Figure 2:**
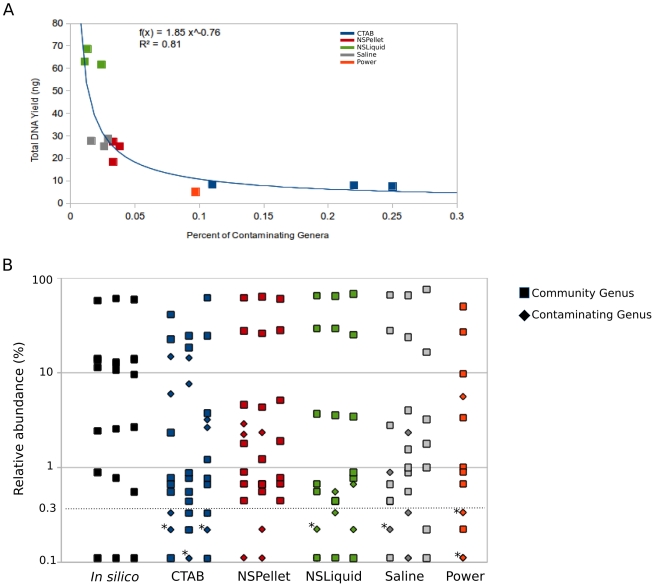
Examination of contaminants in the mock community. (A) Relationship between DNA yield and percent of contaminating genera in the mock community. The equation for a power law regression with coefficient of determination are presented in the inset. (B) Relative abundances of known mock community and spurious (contaminating) genera in mock community profiles. Asterisks indicate data points which represent more than one genus.

Contaminating genera not present in the mock community were also detected in all pyrosequenced samples (**Figure 1** **; Table S3**). Despite the failure of NTCs to produce amplification products (see Methods), these genera were not due to sequencing artifacts as analysis of the in silico communities indicated that sequencing errors were not sufficient to introduce additional genera. Thus, any OTUs with assigned taxonomy outside of the nine genera known to comprise the mock community were considered *bona fide* contaminants. The proportion of contaminants in each library followed a power law relationship with DNA yield, i.e. lower yield was correlated with higher contamination (**Figure 2A**). In general, contaminating genera demonstrated lower relative abundances in the microbial profiles than genera truly belonging to the mock community but this varied substantially between extraction methods (**Figure 2B**). As described above, for 900 sequences, only OTUs present in the community at greater than 0.3% abundance are expected to be detected with 95% confidence. Thus, 0.3% could be used as an empirical cutoff to exclude potentially spurious OTUs. Over half of the contaminating genera appeared at less than 0.3% relative abundance (**Figure 2B** **; Table S3**). A small proportion of expected genera (i.e. non-contaminants) were also present at abundances less than 0.3%.

The PowerSoil and CTAB methods had the highest level of contamination, with an average of ∼10 spurious genera per library comprising approximately 9 and 18 percent of the amplicon libraries respectively (**Table S3**). In particular the CTAB extracted samples had a high percentage of *Stenotrophomonas*. Two contaminants were detected in all extracted samples: members of the genera *Escherichia* and *Dechloromonas* (**Figure 1**). *Escherichia* comprised one 97% OTU which was determined to be *E. coli* by BLASTn analysis (e-value<1e-163). Contamination with this OTU was as high as 7.5% in the CTAB-extracted samples, while the relative abundance of *Dechloromonas* (also represented by a single OTU) was less than 0.01% in all cases (**Figure 1**).

### Reproducibility of DNA extraction methods in the mock community

**Figure 3:**
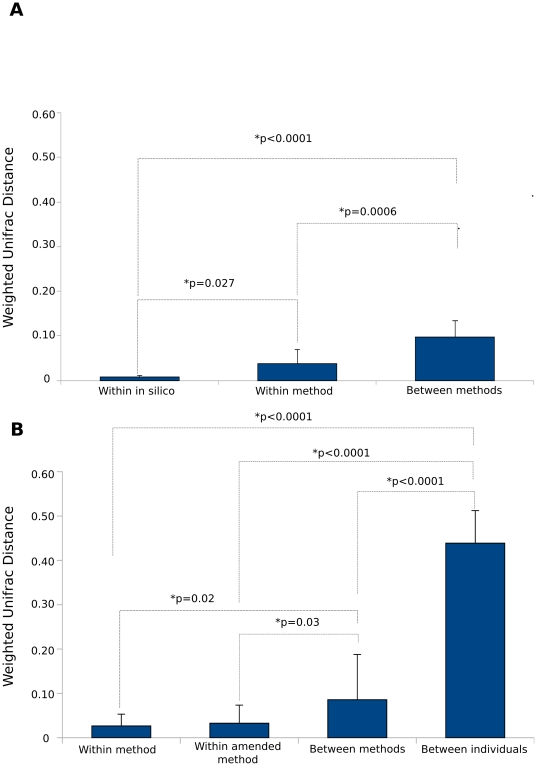
Average weighted Unifrac distances with standard error. Distances for the mock community are presented in (A) and for BAL samples in (B). Significant differences were evaluated using non-parametric exact Mann-Whitney U tests.

Mock community libraries generated using the same DNA extraction method were significantly more similar to each other than to those using different extraction methods (**Figure 3A**). Community composition was compared using weighted Unifrac distances, which account for both community membership and relative abundance [42]. The average Unifrac distance between samples extracted using the same method (technical replicates) was significantly greater than between in silico communities (Mann-Whitney-U test, p-value = 0.027). Between-method distances were significantly greater than both within-in silico and within-method distances, indicating that variation between technical replicates was negligible when compared to differences between methods (p<0.0001). Unifrac distances were largest on average between the PowerSoil method and all other methods (0.651±0.033).

While Unifrac distances between technical replicates were small in general, individual DNA extraction methods varied in terms of technical reproducibility. Unifrac distances were on average an order of magnitude greater between technical replicates of the CTAB method (0.102±0.037) than all other methods. The average Unifrac distance for the saline method (0.029±0.012) was approximately three times greater than for the Nucleospin protocols (NSLiquid: 0.008±0.006, NSPellet: 0.011±0.004), which were comparable to the in silico communities (0.009±0.001).

### Reproducibility of DNA extraction methods in BAL samples

All five extraction methods were tested with at least one replicate in one CF patient (CF356), while only four methods were tested in the other CF patient (CF708) with no technical replication. Replication in the BAL samples was restricted by the volume of BAL fluid available for testing. The CTAB, NSPellet, NSLiquid, and Saline methods were also performed with a dithiothreitol (DTT) pre-treatment in the two CF patients. DTT has been identified as an effective means to liquefy CF sputum samples based on its ability to break disulfide bonds and thus disrupt protein-glycoprotein complexes [43], [44]. The samples from the non-CF patient were extracted using all five methods with technical replication for one method (NSPellet); however the NSLiquid and Saline protocols failed to produce amplifiable DNA. Real-time PCR was used to assess these samples for PCR inhibition and for the presence of both microbial and human DNA. No PCR inhibition was present; however, these two samples contained no detectable microbial DNA and large amounts of human DNA relative to controls (**Figure S2**).

**Figure 4:**
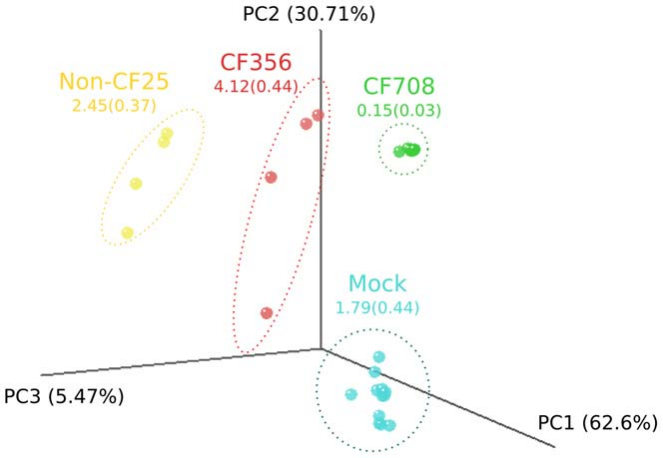
Principal components analysis based on weighted Unifrac distances for BAL samples and mock community extracted using five different extraction methods. CF samples processed with DTT (Sputasol) are not included.

Weighted Unifrac distances were calculated within extraction methods, between extraction methods, and between individuals. Consistent with the results for the mock community, Unifrac distances were significantly greater between extraction methods than within the same method (**Figure 3B**). Distances were also significantly larger between individuals then between or within DNA extraction methods (**Figure 3B**), and samples clustered by individual in principal components analysis (PCA) (**Figure 4**). PERMANOVA analysis based on weighted Unifrac distance indicated a significant effect of individual (p = 0.001), but neither extraction method nor the interaction between individual and extraction method were significant (p = 0.649 and p = 0.885 respectively). The average Unifrac distance between different methods for CF708 (0.005±0.008) were much smaller than for CF356 (0.116±0.029) and non-CF25 (0.168±0.080). The Shannon index indicated correspondingly lower diversity in CF708's microbial community as compared to the other two individuals, suggesting that reproducibility may be higher in lower diversity samples (**Figure 4**).

**Figure 5:**
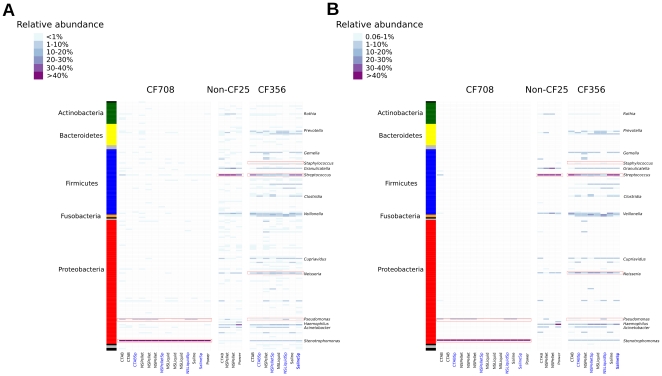
Microbial community profiles for BAL samples. 16 S libraries were normalized to 400 sequences and 97% OTUs were consolidated at the genus level. Red boxes indicate genera previously cultured during routine microbiology. Samples processed with DTT (Sputasol) are labeled in blue. Community profiles including all sequences are presented in (A), and profiles excluding sequences at less than 0.6% relative abundance are presented in (B).

Pre-treatment with DTT (Sputasol) did not significantly change the composition of BAL microbial communities (**Figure 3B** **;** **Figure 5**). Average Weighted Unifrac distances between communities extracted with and without DTT were not significantly greater than distances between technical replicates of the same method (**Figure 3B**). PERMANOVA analysis indicated no significant effect of DTT treatment and no interaction between DTT and DNA extraction method (p = 0.633 and p = 0.478 respectively).

### Microbial ecology of pediatric BAL samples

Community profiles of BAL samples were highly consistent with routine clinical microbiology, with dominant populations reflecting previously cultured isolates (**Figure 5** **; Table S4**). CF708 cultured *Stenotrophomonas* at high CFU counts and *Pseudomonas* at much lower counts (**Table S4**). *Stenotrophomonas* was the most abundant organism in the community profiles for this patient regardless of DNA extraction method (>90% in all cases), with *Pseudomonas* the second most abundant for most extraction methods (0.01–10%; **Figure 5**). Similarly in CF356, *Streptococcus* was the most abundant organism both by culturing and sequencing, with *Neisseria*, *Staphylococcus*, and *Pseudomonas* present in lower relative abundances. *Streptococcus* was the only organism identified by culture from the non-CF patient, and it comprised the largest proportion of the microbial community profiles.

Microbial community profiles for the BAL samples were re-analyzed using an empirical cutoff value to exclude potential contaminants (**Figure 5B**). As described in Methods, libraries from the BAL samples were normalized to 400 sequences for comparison, as sequencing efforts were highly variable (**Table S1**). Based on the relationship determined using simulated data (**Figure S1B**), taxa with relative abundances greater than 0.6% would be expected to be detected with 95% confidence using 400 sequences. Filtering of the BAL profiles using the empirical cutoff value of 0.6% removed many low abundance OTUs, most strikingly for CF708, for whom nearly all of the resultant communities were comprised solely of *Stenotrophomonas* and *Pseudomonas* (**Figure 5B**). *Stenotrophomonas* was also detected at lower abundance in CF356 and the non-CF patient in the unfiltered community profiles (**Figure 5A**). Upon filtering, *Stenotrophomonas* was still present at greater than 1% abundance in all samples from CF356, but was absent from the non-CF profiles regardless of extraction method (**Figure 5B**).

In general, the BAL communities were low diversity as compared to other environments such as the human gut, with Shannon indices comparable to those previously reported by Guss et al. for pediatric CF sputum samples [2]. However, BAL profiles revealed more microbial diversity than culturing alone, including the presence of anaerobic bacteria (**Figure 5**). Both CF356 and non-CF25 showed high abundances of *Granulicatella*, *Prevotella*, and other anaerobes such as *Fusobacterium* and *Veillonella* (**Figure 5**).

## Discussion

Amplicon pyrosequencing is becoming a mainstay for culture-independent community profiling using the 16 S rRNA gene [1]. There are several experimental factors that can influence profiles including sequencing errors [21], primer specificity [45], target region [46], and DNA extraction method [7], [8]. Here we further investigate the effects of DNA extraction method on microbial community profiles. Specifically, the trade-off between detection limit and contamination as well as method reproducibility were evaluated in a mock community of known composition and in pediatric bronchoalveolar lavage (BAL) samples.

### Detection limits and empirical cutoff vales for 16 S pyrosequencing

All DNA extraction methods were first tested on a mock microbial community of known composition comprised of twelve bacterial species representing nine genera. Regardless of extraction method, seven of the nine genera were observed in all samples (**Figure 1**). This included *Staphylococcus* which is notably hard to lyse and has been recovered with varying efficiency by different DNA extraction methods [47], [48]. The two genera which were not ubiquitously detected were those with the lowest predicted relative abundance, suggesting that they may have fallen below detection limits. Previous studies have modelled the sequence coverage necessary to detect an OTU with a given frequency by the Poisson distribution [49], [50] and the normal approximation to the binomial, which provides more conservative estimates (i.e. requiring more sequences) [8]. Using simulated mock microbial communities, we demonstrated that a simple model based on the geometric distribution can be used to provide reasonable estimates for the detection limits of microbial community profiling (**Figure S1; Table S2**). Based on these estimates, the magnitude of reads needed to detect the low abundance genera was several fold higher than is typically generated per sample on the pyrosequencing platform [51].

Genera that were not constituents of the mock community were also detected in the sample profiles, which we infer to be reagent contaminants. NTCs for each extraction method failed to produce amplicon sequences (**Table S1**); however, Champlot et al. determined that many NTCs (>20) must be performed to detect contamination at levels of 20 percent or less [52]. The degree of contamination in the sequenced mock community samples was inversely correlated with DNA yield (**Figure 2A**). This is consistent with the observation that reagent contamination with microbial DNA more significantly impacts samples with low amounts of target DNA [52]–[55]. The CTAB protocol produced the lowest DNA yields and the highest percentage of contaminants, largely attributable to *Stenotrophomonas*, a commonly recognized reagent and water contaminant [56]. Two contaminants, *E. coli* and *Dechloromonas*, were ubiqutious, and thus likely they originated during PCR amplification rather than from reagents used in specific DNA extraction protocols. PCR reagents and especially *Taq* polymerase have repeatedly been identified as sources of contamination in 16 S surveys [52], [54], [57]–[60]. *E. coli* DNA in particular has previously been identified in *Taq* preparations and other reagents [61].

To exclude potentially contaminating taxa while preserving *bona fide* OTUs in community profiles, we used the detection thresholds determined by the geometric distribution as empirical cutoff values. Other studies of microbial diversity have similarly used cutoff values based on either OTU relative abundances or the number of sequences comprising the OTU cluster (e.g. the exclusion of singletons, OTU clusters comprised of only one sequence) [62]–[64]. As predicted, in the sequenced mock community, the majority of component genera were reproducibly detected above the cut-off regardless of extraction method, while only a small proportion of true community genera were excluded. Over half of the putative contaminating genera present in the mock community profiles were excluded using the cutoff. Application of an empirical cutoff to the BAL samples excluded all but two taxa (*Pseudomonas* and *Stenotrophomonas*) for CF708. Notably, *Stenotrophomonas* was eliminated from the profile of NonCF25, but was maintained at low abundance for CF356. While *Stenotrophomonas* was not cultured from the BAL sample of CF356 used in this study, it was cultured at high abundance in a BAL taken six months earlier, corroborating these results. In contrast, the non-CF patient had no clinical history of *Stenotrophomonas* infection, and *Stenotrophomonas* may have been a contaminant in these profiles as found in the CTAB extraction of the mock community.

### Reproducibility of DNA extraction methods

DNA extraction methods varied in their technical reproducibility in both mock and BAL samples. Reproducibility was assessed by comparing weighted Unifrac distances between technical replicates for each method as well as between in silico replicates of the mock community. Technical replication in BAL samples was restricted by sample volume, as in young children, the amount of BAL fluid obtained can be limited due to small starting volumes adjusted for body weight and low lavage fluid recovery rates [65]. In the mock community, the CTAB method was the least reproducible, while between-replicate Unifrac distances for the Nucleospin methods were comparable to the idealized in silico communities. CTAB extractions have previously been shown to be less reproducible than other methods for the extraction of microbial DNA [66]. Kit-based extractions demonstrated less technical variation than organic methods in a metagenomic study of a mock community [9], as the use of pre-made buffers and column purifications likely reduces introduced error. Salonen et al. have suggested that protocols with many steps, such as the CTAB method, may not be appropriate for large-scale studies, and also increase the potential for higher technical variation [67].

In the two CF BAL samples, a subset of the DNA extraction methods were tested with and without the addition of the common mucolytic agent dithiothreitol (DTT) to determine if DTT introduced significant variation in microbial profiles. We did this because amendments to sample processing such as the addition of glycerol have been shown in some instances to lead to marked changes in microbial metagenomes [9]. Our results indicate that DTT treatment does not significantly alter microbial community profiles in pediatric BAL samples. DTT treatment has also been shown to have no significant effect on macrophage antigen expression in BAL samples [44].

Weighted Unifrac distances between DNA extraction methods were significantly greater than between technical replicates (and amended replicates) in both the mock and BAL samples (**Figure 3**). Studies of gut microbiota using the 16 S rRNA gene have demonstrated similarly minimal variation between technical replicates versus significantly larger community differences between extraction methods [7], [8], [67], [68]. In fecal and colon biopsy samples, observed community differences between extraction methods were partly driven by fluctuations in the relative abundance of hard-to-lyse organisms such as Archaea and *Firmicutes* because DNA extraction methods varied in their efficacy in lysing more recalcitrant cell walls [7], [8], [67], [68]. Bead-beating methods in particular significantly increased the proportion of *Firmicutes* in 16 S microarray profiles [67], [68]. In our mock community samples, the largest weighted Unifrac differences were noted between PowerSoil and all other extraction methods. Some of this difference was attributable to the presence of contaminants as discussed above; however, the PowerSoil extraction demonstrated the best recovery of *Staphylococcus* and *Streptococcu*s as compared to all other methods. The recovery of *Staphylococcus* was also enriched in one of the BAL samples (Non-CF25) as compared to other methods. PowerSoil is the only protocol in the present study which includes a bead-beating step while all others use enzymatic and chemical lysis (**Table 1**). Mechanical lysis is likely more effective in disrupting Gram-positive bacteria and other hard-to-lyse organisms [47], [69].

Regardless of which DNA extraction method was used on BAL samples, individual patients retained diagnostic profiles that uniquely identified them. Weighted Unifrac distances between individuals were on average four times greater than between extraction methods (**Figure 3B**). Comparison of DNA extraction methods in studies of gut microbiota also demonstrated large inter-individual community differences, with smaller variations due to methodological differences [7], [8], [70]. Momozawa et al. reported Unifrac distances that were threefold greater between individuals than between extraction methods for fecal and colon biopsy samples, which is comparable to our results for BAL samples [8]. It should also be noted that the BAL samples used in this study were frozen raw and stored at −80°C for over five years prior to analysis. For CF sputum samples, it was recently shown that differences in community profiles introduced by storage at different temperatures were insignificant when compared to differences between individual samples [70]. Microbial community profiles of fecal, skin, and soil samples showed a similar lack of variation due to storage temperatures and conditions [7], [71].

### Microbial community profiles of pediatric BAL samples

BAL community profiles were consistent with historical culturing results obtained at the time of BAL acquisition. Recent studies of both CF sputum and lung tissue have demonstrated a high concordance between culturing and 16 S sequencing for identification of the dominant microbial taxa in respiratory samples from CF patients [2], [72]. This is in striking contrast to environmental samples and systems where the dominant isolate rarely represents the most abundant member of the community [73]. The high concordance with culture data suggests that frozen storage does not dramatically alter the composition of the microbial community in pediatric BAL samples, as demonstrated for other human microbiome samples and discussed above [7], [70], [71].

In addition to previously cultured bacteria, community profiling identified a number of anaerobic genera that are gaining acceptance as constituents of the respiratory microbiome. Routine microbiological culture generally does not include anaerobic cultivation, which results in these organisms remaining undetected. Culture-independent studies have demonstrated the presence of organisms not typically detected by culture in pediatric CF BAL samples, including a high prevalence of *Prevotella* and *Granulicatella* species [2], [4], [74]. While *Granulicatella* is not an obligate anaerobe, it can be difficult to detect in culture because it has complex growth requirements and often presents as small satellite colonies adjacent to other *Streptococcus* species [75]. It is still uncertain whether anaerobes actively contribute to disease or are merely passive constituents of transient or resident microbiota, as they have also been implicated as members of the healthy respiratory microbiome [6]. However, *Granulicatella* spp. have been linked with endocarditis and some *Fusobacterium* species have been associated with colorectal cancer [75], [76], suggesting that they may have analogous pathogenic roles in the respiratory tract.

In conclusion, we have shown using simulated and sequenced mock microbial communities that the geometric distribution may provide a useful guide for selecting an empirical cut-off value that optimizes the trade off between detecting real OTUs and filtering out spurious OTUs. Our results indicated that the use of empirical cutoffs may help to exclude contaminating OTUs from microbial profiles, however, at the cost of excluding true community members present at low abundance. Future studies will need to increase sequencing effort to capture low abundance taxa in community profiles. Comparison of DNA extraction methods in the mock and BAL communities indicated that differences between technical replicates of the same extraction method were negligible as compared to differences between methods, emphasizing the need to standardize methodology for sample series. Despite these differences, community profiles in the BAL samples were unique to each individual and were consistent with culturing results from the time of BAL acquisition. Community profiling also identified several anaerobes in the BAL samples that may be active members of the respiratory microbiome. These results should help researchers formulate sampling, extraction and analysis strategies for respiratory and other human microbiome samples.

## Supporting Information

Figure S1 **Modeling of detection limits using the geometric distribution.** (A) Empirical and theoretical cumulative probability distributions for taxa in the mock community. Theoretical distributions were calculated as the geometric cumulative probability using the taxon relative abundance as an estimate for the parameter p. Empirical distributions were calculated using the results of a simulation. *Haemophilus* and *Burkholderia* had expected relative abundances very similar to Staphylococcus and thus are not shown. The blue dotted line demonstrates the level of sequencing necessary to detect a taxon with 95% confidence. (B) Number of sequences necessary for detection at 95% confidence as a function of relative abundance in the simulated mock community. A power law regression was fit to the data, and is shown by the blue dotted line. The green dotted line represents 900 sequences, and the red dotted line represents 400 sequences.
(PDF)

Figure S2 **Normalized real-time PCR data for a subset of non-CF25 samples.** Axes show 2∧deltaCT values: CT values for 16 S real-time assay were normalized to the non-human control (NHC), while CT values for the human ERV-3 real-time assay were normalized to the non-microbial control (NMC). A non-template control (NTC) is provided for comparison.
(PDF)

Table S1 Number of sequences in in silico and 454 amplicon libraries following Acacia correction, and length and quality filtering.
(DOC)

Table S2 Predicted relative abundance of genera in the simulated mock communities, Komologorov-Smirnov (KS) p-values and 95% confidence sequence cutoffs for detection. The predicted relative abundances were calculated by Grinder by adjusting the input relative abundance and adjusting for copy number bias. The KS test was used to determine whether the empirical cumulative probability distribution for each genus was significantly different from the theoretical geometric distribution. The sequence cutoff is the number of sequences necessary to detect a taxon at the given relative abundance with 95% confidence based on the geometric distribution.
(DOC)

Table S3 Average number of genera detected in mock community samples by extraction method with standard deviation with and without relative abundance threshold.
(DOC)

Table S4 Organisms cultured from BAL samples at time of acquisition with colony forming unit (CFU) counts per mL of BAL fluid.
(DOC)
