# Supplementary figures and images for: Comparison of DNA Extraction Methods for Microbial Community Profiling with an Application to Pediatric Bronchoalveolar Lavage Samples
Source: PLoS One. 2012 Apr 13;7(4):e34605. doi: 10.1371/journal.pone.0034605 (PMC3326054; doi:10.1371/journal.pone.0034605)

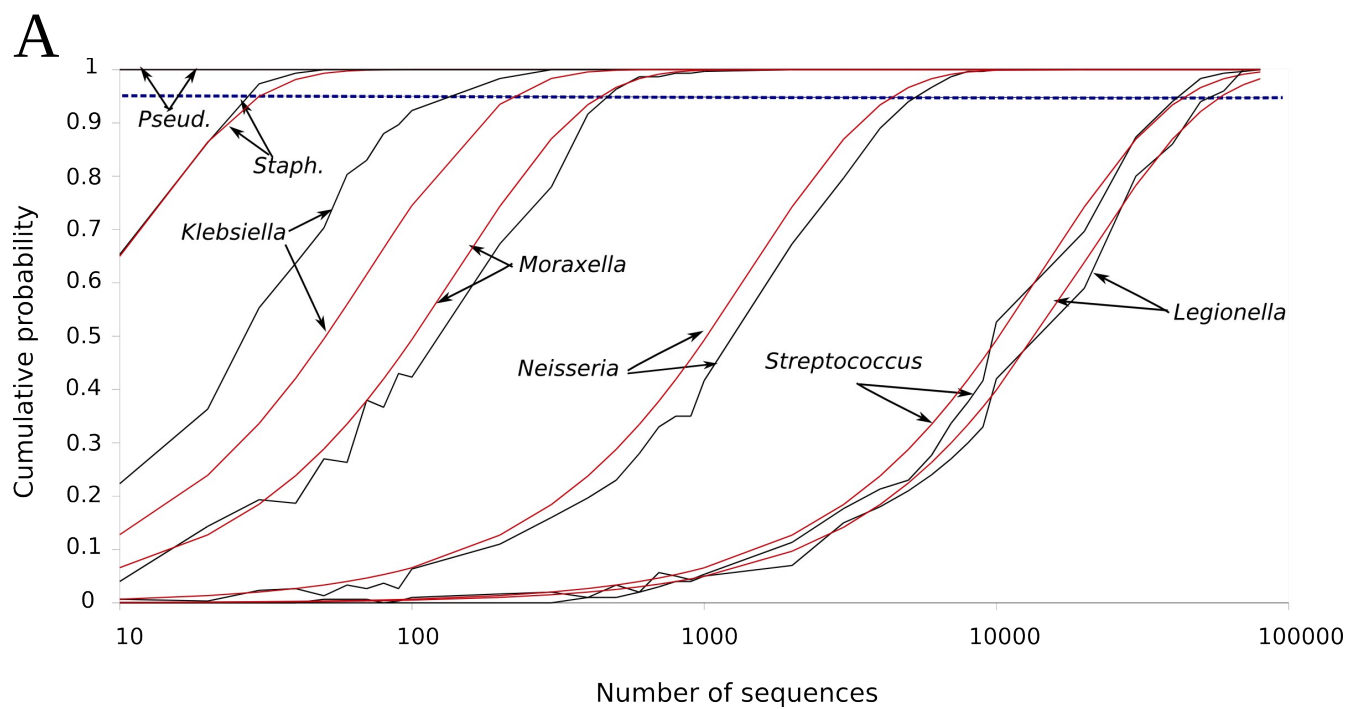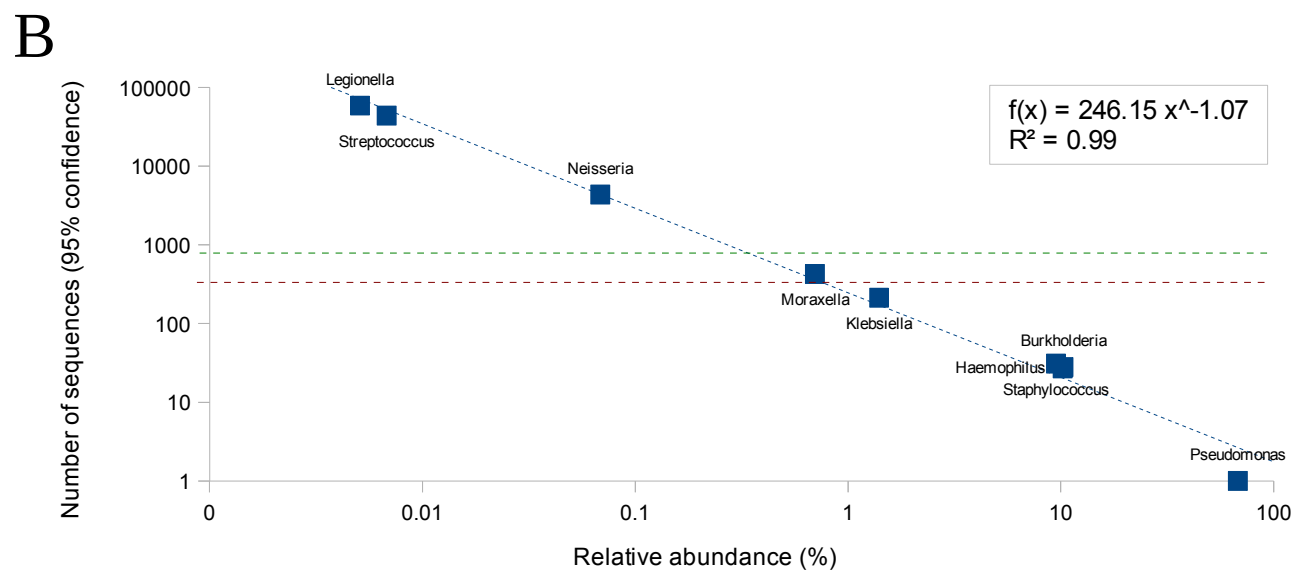

Supplement: Figure S1 — Modeling of detection limits using the geometric distribution. (A) Empirical and theoretical cumulative probability distributions for taxa in the mock community. Theoretical distributions were calculated as the geometric cumulative probability using the taxon relative abundance as an estimate for the parameter p. Empirical distributions were calculated using the results of a simulation. Haemophilus and Burkholderia had expected relative abundances very similar to Staphylococcus and thus are not shown. The blue dotted line demonstrates the level of sequencing necessary to detect a taxon with 95% confidence. (B) Number of sequences necessary for detection at 95% confidence as a function of relative abundance in the simulated mock community. A power law regression was fit to the data, and is shown by the blue dotted line. The green dotted line represents 900 sequences, and the red dotted line represents 400 sequences. (PDF) [file pone.0034605.s001.pdf]

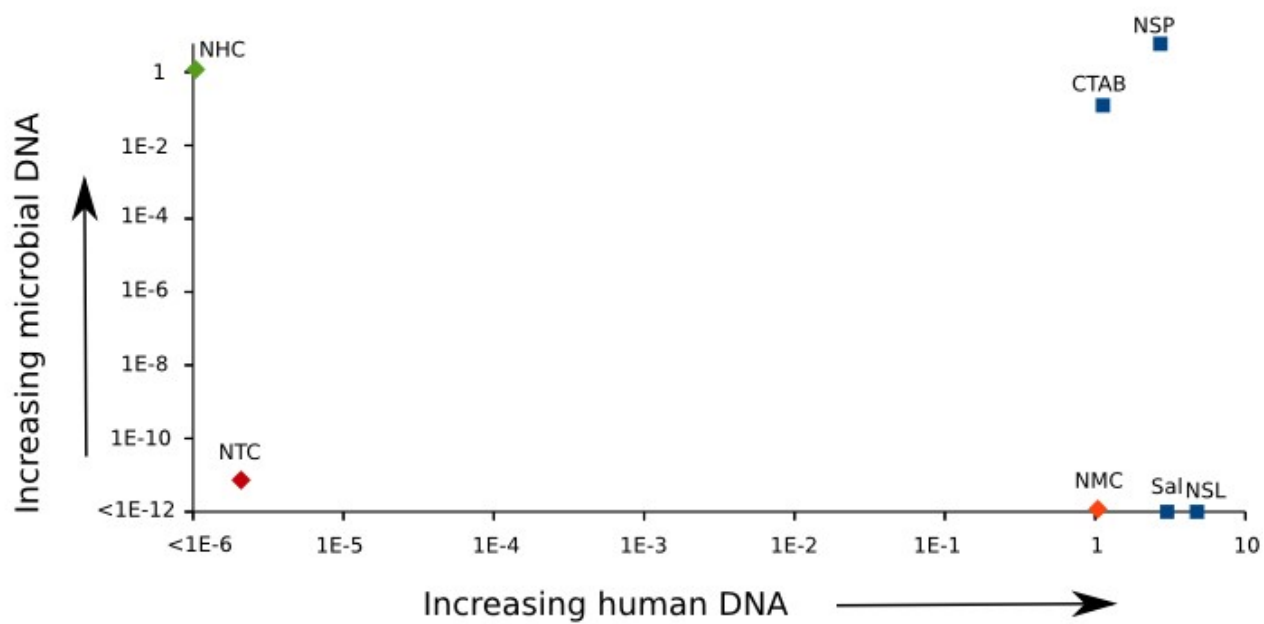

Supplement: Figure S2 — Normalized real-time PCR data for a subset of non-CF25 samples. Axes show 2∧deltaCT values: CT values for 16 S real-time assay were normalized to the non-human control (NHC), while CT values for the human ERV-3 real-time assay were normalized to the non-microbial control (NMC). A non-template control (NTC) is provided for comparison. (PDF) [file pone.0034605.s002.pdf]
