# Supplementary material for: Comparison of DNA Extraction Methods for Microbial Community Profiling with an Application to Pediatric Bronchoalveolar Lavage Samples
Source: PLoS One. 2012 Apr 13;7(4):e34605. doi: 10.1371/journal.pone.0034605 (PMC3326054; doi:10.1371/journal.pone.0034605)
Supplement: Table S1 — Number of sequences in in silico and 454 amplicon libraries following Acacia correction, and length and quality filtering. (DOC) [file pone.0034605.s003.doc]

| Library Name | Number of sequences | | |
| --- | --- | --- | --- |
|  | Replicate 1 | Replicate 2 | Replicate 3 |
| Mock in silico | 3310 | 3292 | 3286 |
| Mock CTAB | 1057 | 987 | 1768 |
| Mock NSPellet | 5839 | 2848 | 5998 |
| Mock NSLiquid | 1874 | 7584 | 1785 |
| Mock Saline | 2995 | 3083 | 2909 |
| Mock PowerSoil | 2189 | - | - |
| CF708 CTAB | 4365 | 2020 | - |
| CF708 CTAB Sputasol | 1655 | - | - |
| CF708 NSPellet | 2911 | 5465 | 2749 |
| CF708 NSPellet Sputasol | 2572 | - | - |
| CF708 NSLiquid | 1960 | 5957 | 3413 |
| CF708 NSLiquid Sputasol | 1171 | - | - |
| CF708 Saline | 2679 | - | - |
| CF708 Saline Sputasol | 2311 | - | - |
| CF708 PowerSoil | 2746 | - | - |
| CF356 CTAB | 1162 | - | - |
| CF356 CTAB Sputasol | 1784 | - | - |
| CF356 NSPellet | 2016 | - | - |
| CF356 NSPellet Sputasol | 3117 | - | - |
| CF356 NSLiquid | 1231 | - | - |
| CF356 NSLiquid Sputasol | 445 | - | - |
| CF356 Saline | 1159 | - | - |
| CF356 Saline Sputasol | 4180 | - | - |
| Non-CF25 CTAB | 1810 | - | - |
| Non-CF25 NSPellet | 2554 | 1279 | - |
| Non-CF25 PowerSoil | 1570 | - | - |
| NTC CTAB | 0 | - | - |
| NTC NSPellet | 0 | - | - |
| NTC PowerSoil | 0 | - | - |
| NTC Saline | 0 | - | - |
