# Supplementary material for: Comparison of DNA Extraction Methods for Microbial Community Profiling with an Application to Pediatric Bronchoalveolar Lavage Samples
Source: PLoS One. 2012 Apr 13;7(4):e34605. doi: 10.1371/journal.pone.0034605 (PMC3326054; doi:10.1371/journal.pone.0034605)
Supplement: Table S2 — Predicted relative abundance of genera in the simulated mock communities, Komologorov-Smirnov (KS) p-values and 95% confidence sequence cutoffs for detection. The predicted relative abundances were calculated by Grinder by adjusting the input relative abundance and adjusting for copy number bias. The KS test was used to determine whether the empirical cumulative probability distribution for each genus was significantly different from the theoretical geometric distribution. The sequence cutoff is the number of sequences necessary to detect a taxon at the given relative abundance with 95% confidence based on the geometric distribution. (DOC) [file pone.0034605.s004.doc]

| Taxon | Relative abundance | KS-test p-value | 95% confidence sequence cutoff |
| --- | --- | --- | --- |
| *Pseudomonas* | 0.679 | 0.960 | 1 |
| *Burkholderia* | 0.103 | 0.320 | 28 |
| *Haemophilus* | 0.102 | 0.486 | 0 |
| *Staphylococcus* | 0.095 | 1.000 | 31 |
| *Klebsiella* | 0.014 | 0.063 | 213 |
| *Moraxella* | 0.007 | 1.000 | 427 |
| *Neisseria* | 6.85E-004 | 0.854 | 4,372 |
| *Streptococcus* | 6.79E-005 | 0.867 | 44,118 |
| *Legionella* | 5.09E-005 | 0.320 | 58,854 |
