# Supplementary material for: Comparison of DNA Extraction Methods for Microbial Community Profiling with an Application to Pediatric Bronchoalveolar Lavage Samples
Source: PLoS One. 2012 Apr 13;7(4):e34605. doi: 10.1371/journal.pone.0034605 (PMC3326054; doi:10.1371/journal.pone.0034605)
Supplement: Table S3 — Average number of genera detected in mock community samples by extraction method with standard deviation with and without relative abundance threshold. (DOC) [file pone.0034605.s005.doc]

|  | **No threshold** | | | | **Relative abundance threshold of 0.003** | | |
| --- | --- | --- | --- | --- | --- | --- | --- |
|  | Total genera | Community genera | Contaminant genera | Contaminant total relative abundance (%) | Total genera | Community genera | Contaminant genera |
| In silico | 7.00 (0) | 7.00 (0) | 0 (0) | 0 (0) | 6.00 (0) | 6.00 (0) | 0 (0) |
| CTAB | 17.7 (3.79) | 8.00 (0) | 9.67(3.79) | 18.8 (6.57) | 9.67 (0.58) | 6.33 (0.58) | 3.67 (0.58) |
| NSPellet | 9.00 (0) | 7.00 (0) | 2.00 (0) | 3.48 (0.26) | 8.00(0) | 7.00 (0) | 1.00 (0) |
| NSLiquid | 9.33 (0.58) | 7.00 (0) | 2.33 (0.58) | 1.62 (0.71) | 6.67 (0.58) | 5.67 (0.58) | 1.00 (0) |
| Saline | 11.3 (0.58) | 7.33 (0.58) | 4.00 (1) | 2.33 (0.69) | 7.67 (1.53) | 6 (1.00) | 1.67 (0.58) |
| PowerSoil | 18.0 | 8.00 | 10.0 | 9.67 | 10.0 | 7.00 | 3.00 |
