# Supplementary material for: Comparison of DNA Extraction Methods for Microbial Community Profiling with an Application to Pediatric Bronchoalveolar Lavage Samples
Source: PLoS One. 2012 Apr 13;7(4):e34605. doi: 10.1371/journal.pone.0034605 (PMC3326054; doi:10.1371/journal.pone.0034605)
Supplement: Table S4 — Organisms cultured from BAL samples at time of acquisition with colony forming unit (CFU) counts per mL of BAL fluid. (DOC) [file pone.0034605.s006.doc]

| Individual | Organism | CFUs/mL |
| --- | --- | --- |
| CF708 | *Stenotrophomonas maltophilia* | 100,000,000 |
|  | *Pseudomonas aeruginosa* | 500,000 |
| CF356 | S*treptococcus* non-hemolytic | 200,000 |
|  | *Streptococcus* alpha-hemolytic | 90,000 |
|  | *Neisseria spp.* | 90,000 |
|  | *Staphylococcus* | 10,000 |
|  | *Pseudomonas spp.* | 1,000 |
| Non-CF25 | *Streptococcus* alpha-hemolytic | 50,000 |
|  | Normal respiratory flora | 100 |
